# Cardiac imaging in athlete’s heart: current status and future prospects

**DOI:** 10.1186/s12947-023-00319-3

**Published:** 2023-12-14

**Authors:** Nurmakhan Zholshybek, Zaukiya Khamitova, Bauyrzhan Toktarbay, Dinara Jumadilova, Nail Khissamutdinov, Tairkhan Dautov, Yeltay Rakhmanov, Makhabbat Bekbossynova, Abduzhappar Gaipov, Alessandro Salustri

**Affiliations:** 1 School of Medicine, Department of Medicine, Nazarbayev University, Astana, 01000 Kazakhstan; 2 National Research Cardiac Surgery Center, Radiology Unit, Astana, 01000 Kazakhstan; 3 National Research Cardiac Surgery Center, Cardiology Unit #2, Astana, 01000 Kazakhstan; 4 Clinical and Academic Department of Radiology and Nuclear Medicine, CF “University Medical Center”, Astana, 01000 Kazakhstan

**Keywords:** Athlete’s heart, Sports cardiology, Cardiac imaging, Hemodynamic forces

## Abstract

**Background:**

Physical activity contributes to changes in cardiac morphology, which are known as “athlete’s heart”. Therefore, these modifications can be characterized using different imaging modalities such as echocardiography, including Doppler (flow Doppler and Doppler myocardial imaging) and speckle-tracking, along with cardiac magnetic resonance, and cardiac computed tomography.

**Main text:**

Echocardiography is the most common method for assessing cardiac structure and function in athletes due to its availability, repeatability, versatility, and low cost. It allows the measurement of parameters like left ventricular wall thickness, cavity dimensions, and mass.

Left ventricular myocardial strain can be measured by tissue Doppler (using the pulse wave Doppler principle) or speckle tracking echocardiography (using the two-dimensional grayscale B-mode images), which provide information on the deformation of the myocardium.

Cardiac magnetic resonance provides a comprehensive evaluation of cardiac morphology and function with superior accuracy compared to echocardiography. With the addition of contrast agents, myocardial state can be characterized. Thus, it is particularly effective in differentiating an athlete’s heart from pathological conditions, however, is less accessible and more expensive compared to other techniques.

Coronary computed tomography is used to assess coronary artery anatomy and identify anomalies or diseases, but its use is limited due to radiation exposure and cost, making it less suitable for young athletes.

A novel approach, hemodynamic forces analysis, uses feature tracking to quantify intraventricular pressure gradients responsible for blood flow. Hemodynamic forces analysis has the potential for studying blood flow within the heart and assessing cardiac function.

**Conclusions:**

In conclusion, each diagnostic technique has its own advantages and limitations for assessing cardiac adaptations in athletes. Examining and comparing the cardiac adaptations resulting from physical activity with the structural cardiac changes identified through different diagnostic modalities is a pivotal focus in the field of sports medicine.

**Graphical Abstract:**

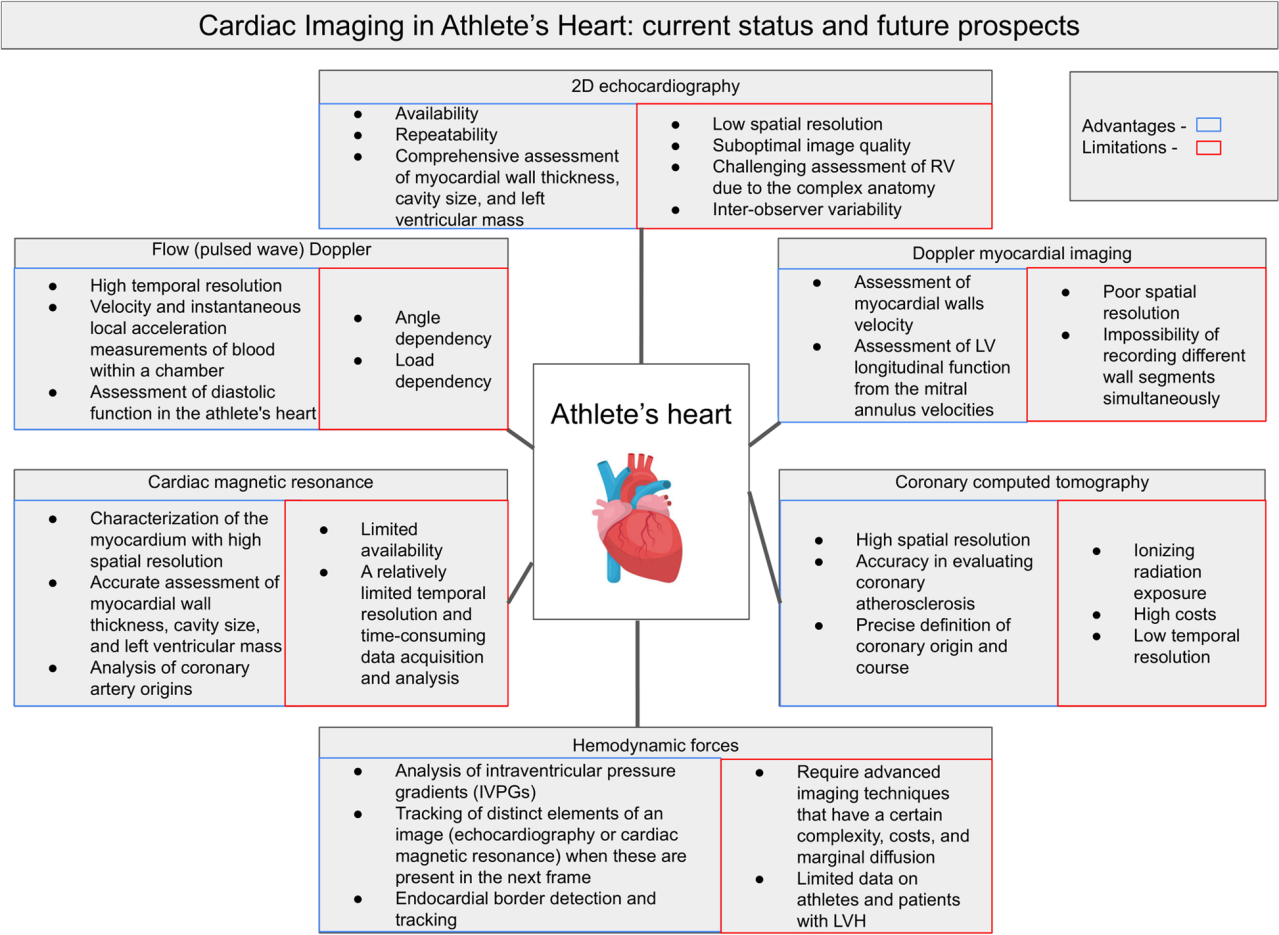

**Supplementary Information:**

The online version contains supplementary material available at 10.1186/s12947-023-00319-3.

## Background

Athletic training induces cardiac adaptations, including morphologic changes of the left ventricle (LV) [1]. The spectrum of such changes after regular and long-term intense physical activity is known as the “athlete’s heart”, characterized by increased mass, cavity dimensions, and wall thickness with at least normal systolic and diastolic function [2], which can be demonstrated by cardiac imaging [3]. The type of cardiac remodeling depends on the type of exercise performed and is represented by two different morphological forms of the athlete’s heart that may be distinguished: strength-trained heart and endurance-trained heart [4]. In the last decades, significant steps have been made in the qualitative assessment of cardiac performance and adaptations in athletes, and several tools can be used to describe such changes, including 2-dimensional (2D) echocardiography, Doppler [including flow Doppler and Doppler myocardial imaging (DMI)], speckle-tracking echocardiography (STE), cardiac magnetic resonance (CMR), and cardiac computed tomography (CCT). All these methods help to distinguish and differentiate physiological adaptations to physical activity in athletes from pathological changes in patients with heart diseases. The relative advantages and drawbacks of each method are summarized in Table 1.

**Table 1:** Common imaging diagnostic methods for cardiac evaluation in athletes

Tools	Advantages	Limitations
2D echocardiography	Availability Repeatability Comprehensive assessment of myocardial wall thickness, cavity size, and left ventricular mass	Low spatial resolution Suboptimal image quality Challenging assessment of right ventricle due to the complex anatomy Inter-observer variability
Doppler		
Flow (pulsed wave) Doppler	High temporal resolution Velocity and instantaneous local acceleration measurements of blood within a chamber Assessment of diastolic function in the athlete's heart	Angle dependency Load dependency
Doppler myocardial imaging	Assessment of myocardial walls velocity Assessment of left ventricular longitudinal function from the mitral annulus velocities	Poor spatial resolution Impossibility of recording different wall segments simultaneously
Cardiac magnetic resonance	Detailed characterization of the myocardium with high spatial resolution Accurate assessment of myocardial wall thickness, cavity size, and left ventricular mass Right ventricular assessment Analysis of coronary artery origins	Limited availability Expensive compared to echocardiography Not feasible in some patients A relatively limited temporal resolution and time-consuming data acquisition and analysis
Cardiac computed tomography	High spatial resolution Accuracy in evaluating coronary atherosclerosis Precise definition of coronary origin and course	Ionizing radiation exposure High costs Low temporal resolution

Currently, there is a lack of clarity regarding the most effective strategies to emphasize the critical characteristics of the athlete’s heart despite the availability of numerous diagnostic techniques [5]. Therefore, the present manuscript highlights the different approaches to diagnosing the athlete’s heart and the main articles on this topic are summarized in Table 2.

**Table 2:** Articles on the assessment of athlete’s heart using different imaging modalities

References №	Study population	Method(s)	Parameters	Main findings	Conclusions
Reference № 1	15 untrained, young, healthy control subjects 20 professional athletes of the same age (mean: 26 years) and sex (male)	2D echocardiography DMI	Right ventricular long-axis dimension LV short-axis dimension Peak systolic velocities S/E and E/A ratios	Athletes had significantly greater right ventricular long-axis and LV short-axis dimensions than the control subjects LV ejection fraction was similar in the two groups Peak systolic velocities significantly increased along the LV short and right ventricular long-axes in athletes	DMI can provide valuable indications for the athlete's heart Velocities, or rather peak velocities, are the most immediate and readable parameters provided by this technique
Reference № 7	947 elite, highly trained athletes who participate in a wide variety of sports	2D echocardiography ECG	LV wall thickness LV end-diastolic dimension Ventricular septal thickness Posterior free-wall thickness LV mass LV mass index Aortic root dimension Left atrial dimension	The thickest LV wall among the athletes measured 16 mm Wall thickness compatible with the diagnosis of hypertrophic cardiomyopathy (≥ 13 mm) was identified in only 16 of the 947 athletes (1.7%)	A LV wall thickness of ≥ 13 mm is uncommon in highly trained athletes and associated with an enlarged LV cavity
Reference № 23	18 male top-level athletes, who were members of the Italian Olympic rowing team (3 consecutive years of long-term exercise) 12 untrained sedentary male subjects	2D echocardiography 3D echocardiography CMR	LV mass LV end diastolic volume LV end systolic volume LV ejection fraction	LV systolic function was normal in top-level athletes and did not differ from that of controls	3D echocardiography is highly accurate, and the operator dependence is very low
Reference № 25	15 patients with hypertrophic cardiomyopathy 20 competitive top-level athletes 18 sedentary normal subjects	2D echocardiography DMI 2D strain	Global longitudinal strain Regional peak systolic strain	In general, there was no significant difference between the strain values of the athletes and the control group, but in some segments the strain values of the control group were significantly higher than those of the athletes	2D strain is a new simple, rapid, and reproducible method to measure systolic strain from standard 2D images It might be used as an additional tool for a comprehensive cardiac evaluation in trained athletes and hypertrophic cardiomyopathy
Reference № 31	19 athletes 10 untrained control subjects	M-mode echocardiography 2D echocardiography CMR	LV mass LV end diastolic volume	The best correlation between CMR and echocardiographic LV mass and volumes was observed using the American Society of Echocardiography 2D echocardiographic method	The American Society of Echocardiography 2D echocardiographic approach, when using CMR as a reference standard, was the most accurate estimator of LV mass and volumes in both controls and athletes
Reference № 32	9 endurance-trained athletes 8 sedentary subjects	M-Mode and Doppler echocardiography CMR	LV mass Left atrial dimension LV end diastolic volume LV end systolic volume Interventricular septum Posterior wall Fractional shortening E/A ratio	Myocardial free fatty acids uptake is not enhanced in the athlete's heart at rest When studied with CMR, the endurance-trained subjects had increased LV mass, end-diastolic volume, and stroke volume compared with sedentary subjects	Increases in LV mass, long-axis diameter, and volumes, but also posterior wall thickness in endurance-trained athletes, can reliably be observed by CMR
Reference № 33	Endurance- trained athletes (10 males, 10 females) Strength-trained athletes (8 males, 10 females) Untrained subjects (9 males, 10 females)	2D echocardiography CMR	LV dimensions and function VO_2_ max	Endurance training causes an increase in stroke volume and LV mass, irrespective of gender In contrast, the highly strength-trained males and females did not have increased cardiac dimensions or VO_2_ max compared to untrained controls, and no gender difference was seen	Exclusively practicing strength training does not cause any beneficial effects on the oxygen transport chain

## Main text

### Echocardiography

Due to its extensive availability, easy repeatability, versatility, and low cost, echocardiography is undoubtedly the most common method for a comprehensive assessment of cardiac structure and function in athletes and for assessing the effect of training on an athlete’s heart [6, 7]. Accordingly, its main application in athletes relies in differentiating training-induced adaptations from pathologic changes such as hypertrophic (HCM) or dilated cardiomyopathy [8].

**Fig. 1:**
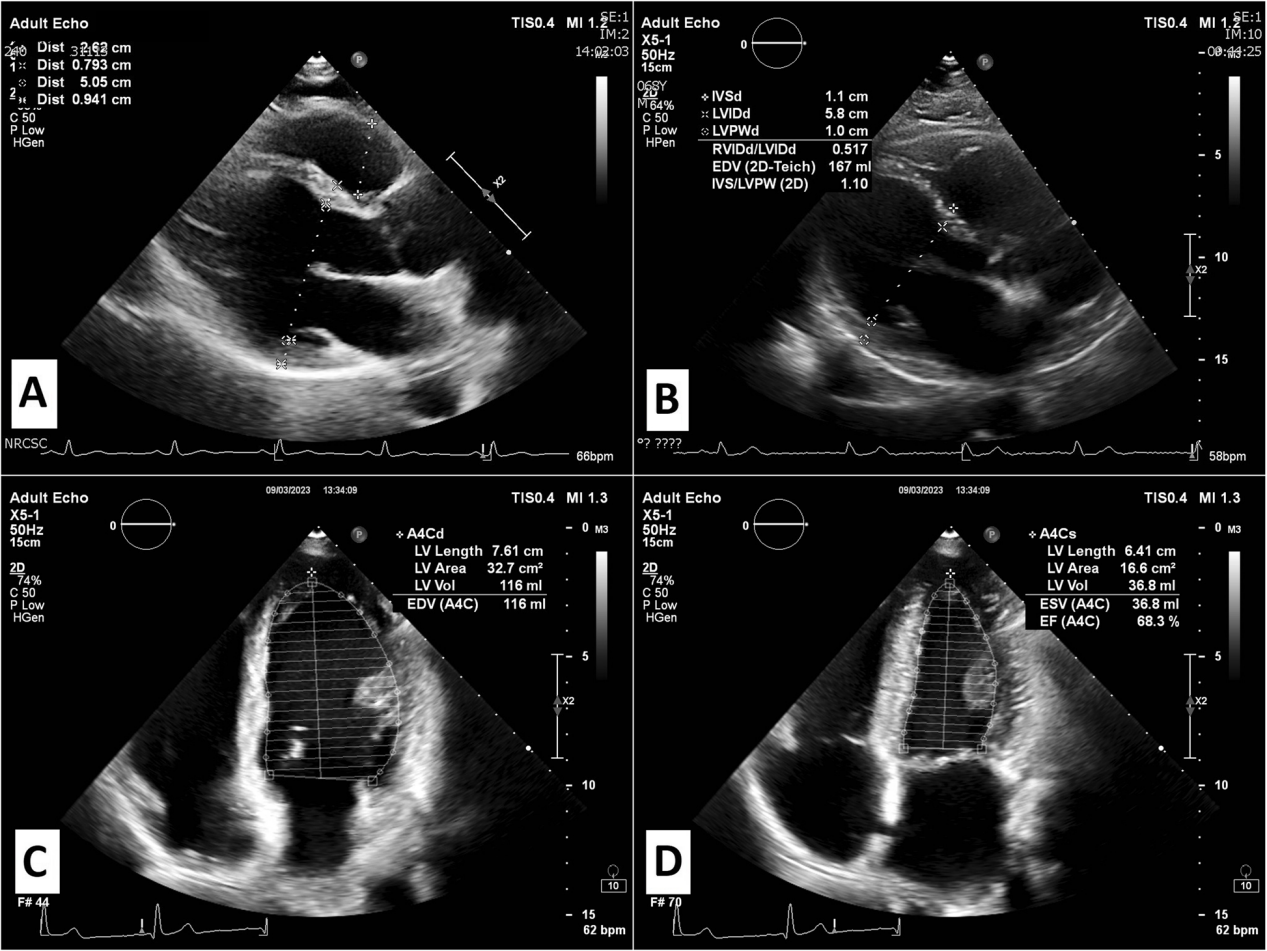
Representation of 2D echocardiography: **A**–**B** Parasternal long-axis view in diastole; **C** Apical 4-chamber view in diastole; **D** Apical 4-chamber view in systole. From these views, detailed assessment of left ventricular dimensions, wall thickness, and volumes can be performed

The advent of echocardiography has allowed us to assess objectively the changes of the cardiac structures in response to different types of exercise [9], and standard 2D echocardiography (Fig. 1) can be applied to identify the presence and severity of left ventricular hypertrophy (LVH) that occurs in athletes [10]. Strictly speaking, the diagnosis of LVH is based on the assessment of LV mass, which requires the geometrical assumption of the LV shape using formulae like the Penn-cube formula. Clearly, this limits the accuracy of the method since small linear error (likely to occur due to the low spatial resolution of the method) translates into cubic errors. In clinical practice, LV wall thickness [and interventricular septum thickness in diastole (IVSd) in particular] is the echocardiographic parameter generally used for evaluating the presence of hypertrophy. Previous echocardiographic studies have demonstrated that advanced physical activity leads to a 15% increase in LV wall thickness and a 10% increase in cavity dimension for both males and females [11]. A large meta-analysis of studies on the athlete’s heart provides ranges of normality for endurance, strength, and combined exercise [4]. The results of this meta-analysis confirm the hypothesis of divergent cardiac adaptations in dynamic and static sports. Left ventricular mass did not differ significantly between the 3 groups of athletes. Of interest, IVSd was, on average, 10.5 (10.1–10.9) cm in endurance athletes vs 11.8 (10.9–12.7) cm in strength athletes vs 8.8 (8.6–9.1) cm in control subjects (*p* < 0.001). Combined endurance- and strength-trained athletes have the highest values of LV diastolic dimension (56.2 mm) compared to endurance (53.7 mm) and strength (52.1 mm) trained athletes. As a consequence, the mean relative wall thickness of endurance-trained athletes was significantly lower than that of strength-trained athletes (0.389 vs 0.442 mm, *P* = 0.006).

A study by Kreso et al. provided useful information on the different echocardiographic characteristics between an athlete’s heart and HCM [12]. Reference values for the echocardiography parameters for athlete’s heart were as follows: IVSd ≤ 11 mm, LVDd ≤ 52 mm, PWd ≤ 10 mm, and LVDs ≤ 36 mm. In contrast, limit values for HCM were IVSd ≥ 13 mm, LVDd ≤ 43 mm, PWD ≥ 12 mm, and LVDs ≤ 29 mm. Thus, a wall thickness exceeding 15 mm, especially in an asymmetric pattern, is more likely to be of pathological origin rather than a result of exercise-induced cardiac remodeling. In cases of LVH within the "gray zone", typically ranging from 13 to 15 mm, distinguishing between exercise-induced remodeling and mild forms of HCM can be challenging. Prescribed detraining and follow-up cardiac imaging to assess for LVH regression may be required when gray zone hypertrophy remains undifferentiated despite a comprehensive diagnostic approach [13]. In a recent small study involving five strength-trained athletes with concentric LVH, significant regression of LV mass and LV wall thickness was observed during the detraining period [14].

Cardiac sex and age differences are present among master athletes. A study by Wooten et al. [15] demonstrated higher LV mass in male than in female athletes (174 ± 44 vs 141 ± 36 g, *p* < 0.01) due to greater end-diastolic interventricular septal, LV posterior wall, and LV basal diameter. However, when indexing to body surface area, LV mass did not differ between sexes. Septal and posterior wall thickness were associated with age for both men (*r* = 0.27, *p* < 0.01) and women (*r* = 0.51, *p* < 0.01), which may explain the finding of a strong positive correlation between age and LV mass index for both groups (*r* = 0.33 and *r* = 0.43, both *p* < 0.01).

Certain ethnic variations in LV remodeling exist among highly-trained athletes of African/Afro-Caribbean (black) and Caucasian (white) descent. Black athletes exhibit a more significant extent of LVH compared to their white counterparts when examined using 2D echocardiography. Consequently, applying conclusions drawn from studies on white athletes may lead to inaccurate diagnosis of HCM in black athletes and up to disqualification from competitive sport in black athletes with LVH ≥ 15 mm [16]. In addition, echocardiography can be used for evaluating the remodeling of LVH in elite athletes after detraining. In a study by Pelliccia et al. [17] on athletes with wall thickness of ≥ 13 mm, after long-term deconditioning maximum wall thickness decreased by 15% and LV mass by 28%. Of note, while wall thickness returned to normal in each athlete, substantial LV dilatation persisted in 20% of athletes. The possibility that this residual LVH, apparently part of the athlete’s heart syndrome, may have future long-term clinical implications in some individuals cannot be excluded with certainty.

Lastly, direct transthoracic examination of the coronary arteries in adults is possible [18] and may become a useful adjunct to the evaluation of athletes.

As for the right ventricle, the evaluation of its size and function is difficult because the conventional 2D methods are not precise enough. This is mainly due to the right ventricular complex anatomy that does not resemble any familiar geometric shape [19].

In addition to the undeniable advantages of this method, there are also limitations, such as variability between observers in measurements of the interventricular septum or the LV posterior wall thickness [7]. This is due to the main limitation of 2D echocardiography represented by the relatively low space resolution and image quality, in particular in subjects with high thoracic impedance [20]. Clearly, measurements on low-quality images are inherently subject to errors.

**Fig. 2:**
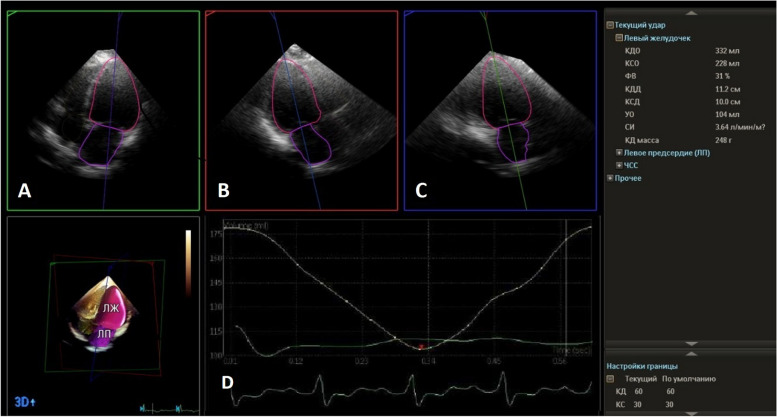
Semi-automated 3D echocardiography. The 3D data set can be analyzed and sliced and 4-chamber (Panel **A**), long-axis (Panel **B**), and 2-chamber views (Panel **C**) can be obtained. Time/volume curves can be automatically displayed (Panel **D**) which provide measurements of LV volumes and ejection fraction

Over the past few decades, there have been significant advancements in ultrasound technology, leading to the evolution of echocardiography from basic M-mode imaging to more advanced techniques such as 3-dimensional (3D) anatomical imaging [21]. The use of 3D echocardiography (Fig. 2) has significantly improved the diagnostic accuracy of cardiac ultrasound in evaluating cardiac anatomy and ventricular function. This approach allows live acquisition of a volumetric dataset that can be analyzed off-line for quantification of LV volume and mass similar to magnetic resonance but with the added benefits of being more cost-effective, repeatable, and suitable for a broader range of people, including athletes [22]. Unlike the 2D approach, 3D echocardiography provides more detailed information on LV remodeling and function and reveals morphological features, such as differences in the shape of the LV chamber, that cannot be adequately assessed using the conventional 2D approach [23].

With all the advantages and disadvantages mentioned above, echocardiography is expected to continue being the first-line imaging modality for assessing the heart of athletes [5].

### Doppler

Flow pulsed wave Doppler echocardiography allows imaging of high-quality signals that provide blood velocity measurements [19]. Based on its characteristics, Doppler technology has led to significant advancements in the study of diastolic function in athletes’ hearts. The majority of cross-sectional studies aimed at identifying differences in LV diastolic function between athletes and non-athletes have focused on the blood flow patterns through the mitral valve [24]. The large meta-analysis already mentioned [4] indicated that the transmitral E/A ratio was not significantly different between athletes and controls, although athletes showed slightly higher values of E/A ratio compared to controls [24].

Doppler myocardial imaging measures the velocity of myocardial walls, which allows the characterization of each ventricular myocardial segment by placing the sample volume at the center of the cardiac wall. Although standard 2D echocardiography is a unique tool in evaluating cardiac adaptations to physical exercise, recent data [10] indicate the efficacy of DMI in analyzing the myocardial systolic and diastolic function of the athlete's heart. In particular, DMI has shown interesting perspectives in assessing trained individuals in several ways, including:

1. Distinguishing between left and right ventricular hypertrophy and pathological conditions
2. Predicting cardiac performance during physical exercise
3. Evaluating the interaction between the two ventricles
4. Analysing myocardial adaptations to various training protocols
5. Identifying specific genotypes associated with cardiomyopathies at an early stage [10].

Moreover, a study [25] aimed at the differentiation between physiological LVH in athletes and pathological LVH in patients with HCM by using DMI proved that the E/e' ratio might also help in distinguishing between physiological LVH and HCM. Also, individuals with morphological mild HCM, including those with normal measurements of mitral valve inflow Doppler, revealed lower systolic and early diastolic velocities compared to athletes, as determined by assessing longitudinal cardiac function with pulsed DMI at the level of the mitral valve annulus.

**Fig. 3:**
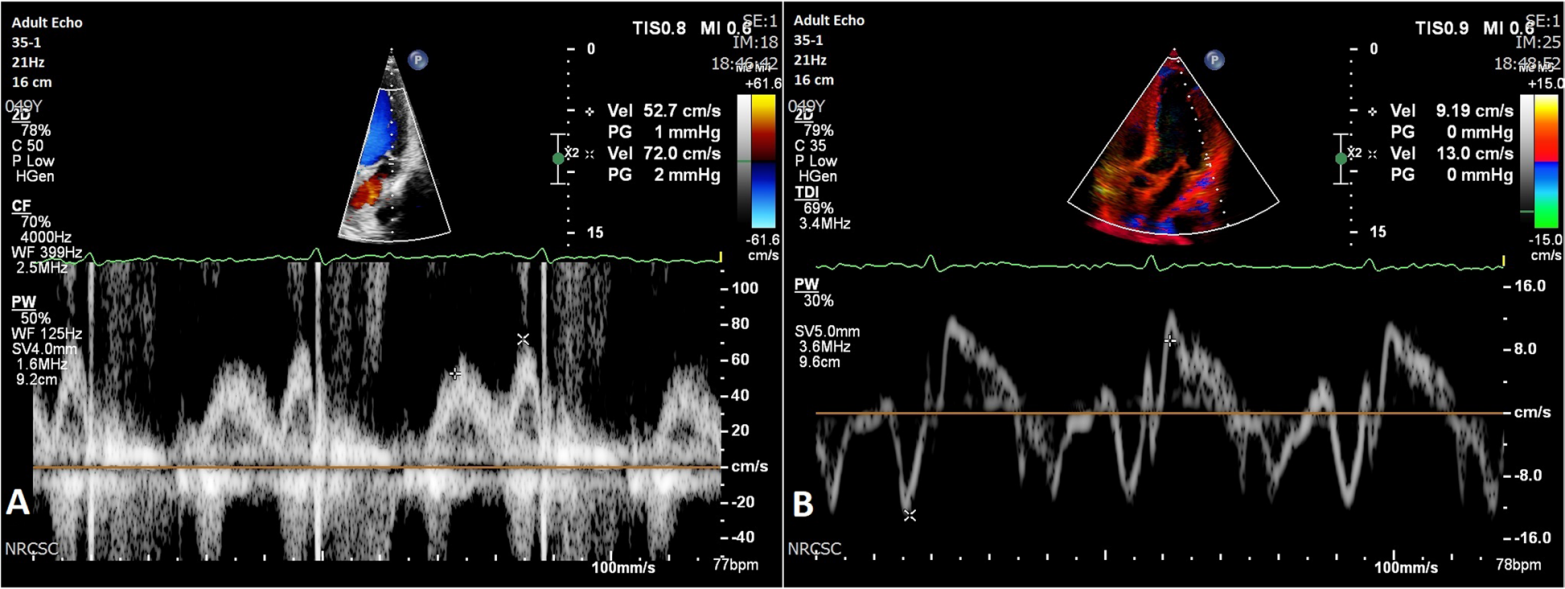
Pulsed wave Doppler echocardiography. Panel **A** represents the spectral velocity profile at the level of the LV inflow. In Panel **B**, the sample volume is located at the level of the lateral mitral annulus, which provides velocity information of the myocardium and evaluation of the LV longitudinal function

Therefore, the combination of standard 2D echocardiography and DMI may be a suitable approach for accurately evaluating both physiological and pathological LVH in a noninvasive and easily repeatable manner [10]. Nonetheless, the limitations of DMI include poor spatial resolution, the challenge of recording distinct wall segments simultaneously [19], and the requirement of precise alignment with the ultrasound beam [21]. A comparative illustration of the two Doppler techniques is represented in Fig. 3.

### Speckle-tracking echocardiography

Speckle-tracking echocardiography is a relatively recent echocardiographic technique of deformation imaging that has provided new insights into the characterization of the athletes’ myocardial properties [21]*.* LV global longitudinal strain (GLS) obtained by STE is the most used parameter in clinical practice, and is not significantly different between athletes and healthy controls. Therefore, a reduction in longitudinal strain in athletes has to be considered as a subclinical sign of LV contractile dysfunction and should raise the suspicion of myocardial disease, particularly in the presence of doubtful LV hypertrophy or dilatation [26].

LV twist from helically oriented fibers is a crucial component of myocardial performance and can be determined by STE. In a number of studies, twist and apical rotation have been found to be lower in athletes (endurance, swimmers, cyclists, soccer players) compared to controls [27]. While the evidence suggests that twist appears to be lower in endurance athletes, some data would suggest that an increase in a twist in endurance (marathon runners) and mixed-trained (martial arts) athletes is a normal phenomenon. Furthermore, Kovàcs et al. evaluated untwist and untwist rate in athletes, HCM, and healthy sedentary volunteers [28]*.* The highest values were measured in athletes, while the lowest were found in HCM (untwist: 51.3 ± 19.1 vs 11.6 ± 10.4 vs 35.9 ± 16.3%; untwist rate: − 32.5 ± 13.0 vs − 10.6 ± 10.8 vs − 23.0 ± 7.7°/s, *p* < 0.05). Thus, there is a potential benefit of untwisting dynamics in differential diagnosis, not only by the use of peak values but also through a temporal assessment.

### Cardiac magnetic resonance imaging

Cardiac magnetic resonance is a crucial tool for providing a comprehensive evaluation of the LV wall thickness and dimensions, and is superior to echocardiography in differentiating an athlete's heart from structural and functional changes due to pathological conditions [29]. Its exceptional tissue contrast, high signal-to-noise ratio allow the acquisition of functional cine CMR sequences, such as steady-state free precession, to clearly identify the epicardial and endocardial borders of the LV walls and accurately measure ventricular wall thickness, regional hypertrophy, and wall motion abnormalities. Cardiac magnetic resonance also provides a reliable assessment of the LV ejection fraction (EF), with no gaps between image slices for precise measurement, and allows for imaging in any plane, including the analysis of coronary origins [30]. In addition, CMR enables accurate assessment of right ventricular volumes, EF, and mass [19]. As a result, CMR has become the reference method for assessing ventricular function.

Multiple studies have compared different echocardiographic approaches with CMR for evaluating LV mass and volumes. These studies consistently found that CMR is a more reliable diagnostic tool than echocardiography due to the absence of geometric assumptions, which is particularly important for distorted left ventricles [31]. For instance, a study by Turpeinen et al. [32] examined LV mass in nine endurance-trained athletes and eight sedentary individuals using CMR and echocardiography. The results showed only a moderate correlation between the two methods (*r* = 0.47, *p* = 0.05). Additionally, when investigating cardiac morphology and function adaptation to endurance and strength training, both volume and mass measurements showed better correlations with the VO_2_ max for CMR-derived LV dimensions than those measured by echocardiography [33].

In addition to morphologic information, CMR is able to detect the presence of myocardial fibrosis using a gadolinium-based contrast agent. The presence of late gadolinium enhancement (LGE), due to extracellular space increase and decreased T1 relaxation time, allows to differentiate between ischemic and non-ischemic (either focal or patchy) patterns of the LGE distribution. Highly trained endurance athletes showed a ten-fold increase in the prevalence of focal LGE as compared to control subjects, always confined to the hinge points [34]. Additionally, those athletes with focal LGE demonstrated globally higher myocardial extracellular volume values. Although a non-specific pattern, this matrix remodeling and potential presence of myocardial fibrosis may be another feature of the athlete’s heart, of which the clinical and prognostic significance remains to be determined.

The insertion point in an athlete’s heart is the most commonly observed pattern, irrespective of age. Its prevalence may reach up to 20% to 30% and has been correlated with a cumulative training load and training intensity [35]. In addition, T1 time and extracellular volume are normal or reduced, while in HCM and dilated cardiomyopathy both are increased. The presence of LGE in locations not observed in athletes or larger areas of LGE, along with an increase in T1-mapping time and extracellular volume, are some of the features that may help to differentiate cardiomyopathy from the athlete’s heart.

Despite its undoubtful advantages for evaluating cardiac morphology and tissue characterisation, CMR is still not widely accessible in many healthcare facilities and is significantly more expensive than echocardiography [36].

### Cardiac computed tomography

The comprehensive evaluation of cardiac morphology made possible by echocardiography and CMR has gained additional advancements with the integration of CCT. In young athletes, this technique offers the highest level of non-invasive resolution for coronary arteries and allows for determining the morphological changes in the coronary arteries (coronary origin and course anomalies). Based on this evidence, predictive information may be provided [37].

Furthermore, in master athletes, CCT (either coronary artery calcium score or coronary computed tomography) is emerging as an outstanding non-invasive imaging technique for evaluating atherosclerosis and is gaining prominence as a vital method for assessing cardiovascular risk and diagnosing coronary artery disease. In this subset of (older) athletes, CCT provides valuable information on the presence of coronary artery disease, calcium score quantification, and cardiovascular risk profile, which might translate into prognostic information. However, the implementation of this imaging technique in pre-participation examination, especially in the case of master endurance athletes, is a matter of current debate.For asymptomatic adults categorized as high risk or very high risk (due to factors such as diabetes, a strong family history of coronary artery disease, or previous risk assessments indicating a high risk for coronary artery disease), the risk assessment should include consideration of a functional imaging test or coronary computed tomography [38].

### New tools for evaluation of cardiac hemodynamics

Hemodynamic forces (HDFs) analysis represents a novel approach to quantifying intraventricular pressure gradients responsible for blood flow. In fact, the functional interplay between the myocardium, valves, and large vessels determines the development of intraventricular pressure gradients, which in turn drive blood flow [39]. Recently, a new noninvasive method based on feature tracking (a technique that analyzes the motion of the naturally occurring pattern in the myocardium) has been proposed.

Left ventricular HDFs can be computed from either echocardiographic or CMR images using offline analysis with dedicated software. Hemodynamic forces are estimated from the same endocardial border tracking used for strain (deformation) calculation. The dimensionless root-means-square of HDFs is computed over the selected period (entire cardiac cycle, systole, or diastole) to measure the overall force amplitude. In order to compare patients with different LV sizes, the HDFs are normalized by the LV volume and expressed as a percentage of gravity acceleration [40]. These normalized forces represent the average pressure gradients in the LV cavity in different directions without directly impacting the volume size.

The following parameters can be calculated in systole, diastole, and over the entire cardiac cycle:

- “Apex-to-base” (A-B, longitudinal) HDFs (%): the normalized entity of HDFs directed in the apex-to-base direction;
- “Latero-septal” (L-S, horizontal) HDFs (%): the normalized entity of HDFs directed in the lateral-septal direction;
- L-S/A-B HDFs ratio (%): the ratio between L-S HDFs and A-B HDFs, used to assess the relative distribution of the HDFs directions in the LV;
- HDFs angle [φ (°)]: the main direction of HDFs over a selected period, using a polar coordinate system. φ ranges from 0°, when the HDFs are directed in an infero-lateral direction, to 360°, after a complete circular turn [41].

**Fig. 4:**
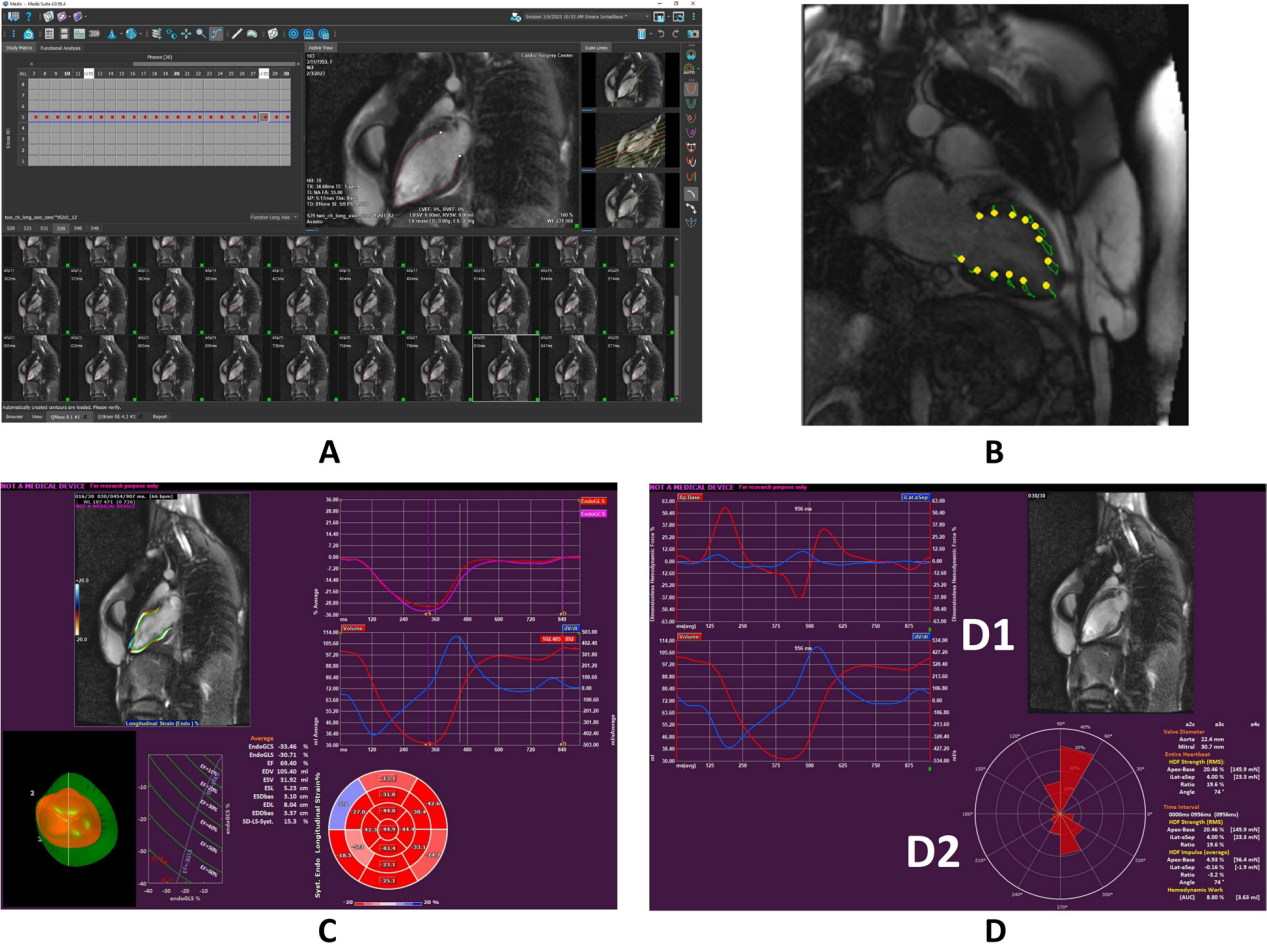
Hemodynamic forces analysis. **A** From CMR dataset, a slice is selected for analysis; **B** Tracing of the LV endocardial border; **C** Strain analysis curves and “bull’s eye” display of myocardial segmental strain. **D** HDFs parameters are represented. Panel D1 shows the Apical-to-Base (red line) and Lateral-to-Septal (blue line) HDFs. Panel D2 shows the polar histogram depicting the distribution and magnitude of LV HDFs (red triangles). In normal subjects, the main HDFs occur in the Apical-to-Base direction

Current data on adaptations of the heart to physical activity in athletes is limited and needs to provide a comprehensive view. To overcome this limitation, HDFs could be used as an alternative to standard methods. Moreover, the feature of HDFs is that computation can be performed from speckle tracking in B-mode echocardiography or feature tracking in cine CMR [42], making this technique available in clinical practice. Figure 4 depicts HDFs performance from cine CMR.

An illustrative example of calculating HDFs from CMR using a feature tracking approach is given in video format with legends in the Supplementary material.

## Conclusions

“Athlete’s heart” refers to specific adaptive changes in the heart that occur as a result of physical activity and contains increased mass, cavity dimensions, and wall thickness with at least normal systolic and diastolic function. Diagnostic techniques such as 2D echocardiography, incorporating Doppler and STE, along with modalities like CMR and CCT, are employed to identify and characterize such modifications. Consequently, each of these methods aids in distinguishing between physiological cardiac adaptations and pathological changes, particularly in situations where uncertainty arises regarding structural cardiac disease.

## Consent for publication

Not applicable.

## Competing interests

The authors declare no competing interests.

### Supplementary Information

**Additional file 1. **Slice selection. Slices (numbered from 1 to 13 in vertical direction) are visually assessed on CMR images shown on the right side panel to choose the most appropriate one for border tracing. Red dots correspond to border tracing and were calculated in this case on two cardiac frames by the software (the remaining frames will be completed by launching the automatic contouring option). For easiness of slice selection on pre-strain analysis page, the observer deletes red dots and leaves only on the slice selected (slice #7) as already traced borders.

**Additional file 2. **Frame selection. Frames (numbered from 1 to 30 in horizontal direction) are visually assessed on the selected slice by observing myocardial contraction/relaxation on CMR images shown on the right side panel to choose those corresponding to end of diastole and end of systole. The observer follows movements of the myocardium and of the mitral valve leaflets throughout the cardiac cycle to identify the end of systole and end of diastole. Once the appropriate frames are identified (in this case the frames #11 and #30), the observer marks them with LV end-systolic and LV end-diastolic frames, respectively.

**Additional file 3. **Border tracing. After slice and frame selection, automatic contouring is performed. In this case, the observer selects both the endo- and epicardial borders to be traced (red and green lines, respectively). When the contouring is finished by the software, the observer visually verifies that all the borders on the selected slice are traced correctly before launching the strain analysis. In the strain analysis results, cine illustration of LV contraction/relaxation shows the direction and the amplitude of the endocardium (see the green lines following the direction of endocardial motion). There are two graphs on the strain analysis page. The upper one presents curves of endocardial global longitudinal (red), circumferential (pink) and radial (green) strain with % average of strain over time at the end of systole. Note that longitudinal and circumferential strain have negative values, while radial strain has positive value. The lower graph shows two curves, where red corresponds to volume (ml average) over time (ms) and blue – to volume change over time change. Minimal LV blood volume at the end of systole corresponds to the lowest point on the curve, while maximal volume – to the highest. Below the graphs, there are two “bull’s eye” diagrams demonstrating systolic myocardial strain percent in longitudinal (red) and transverse (blue) direction with appropriate color assignment to different segments. The cardiac apex is positioned in the centrum of diagrams.

**Additional file 4. **Valve diameter measurement and HDFs analysis. The valve diameter is measured by placing arrows at the annular plane of the valve between internal contours when the leaflets are open: for the mitral valve using two views (4-chamber, then 2-chamber), for the aortic valve – only one view (3-chamber). Once the arrow placement is complete, the software automatically calculates the valve area in the designated box on the right side of the screen. When measurements of both valves are finished, the software readily presents the results of HDFs analysis. The graph on the top represents the Apex-to-Base (A-B, in red) and the Lateral-to-Septal (L-S, in blue) HDFs. The polar histogram on the bottom represents the amplitude and the direction of the HDFs (red triangles). In this case, A-B HDFs are 23.03%, L-S HDFs are 4.02%, and the angle is 71°.

## Data Availability

Not applicable.

## Abbreviations

LV: Left ventricle
2D: 2-Dimensional
DMI: Doppler myocardial imaging
STE: Speckle-tracking echocardiography
CMR: Cardiac magnetic resonance
CCT: Cardiac computed tomography
HCM: Hypertrophic cardiomyopathy
LVH: Left ventricular hypertrophy
IVSd: Intraventricular septum in diastole
LVDd: Left ventricular dimension in diastole
PWd: Posterior wall in diastole
LVDs: Left ventricular dimension in systole
3D: 3-Dimensional
GLS: Global longitudinal strain
EF: Ejection fraction
LGE: Late gadolinium enhancement
HDFs: Hemodynamic forces
